# Primary Assessment of Mycosporine-Like Amino Acids Production by Two Species of *Fischerella *sp.

**DOI:** 10.22037/ijpr.2021.115100.15194

**Published:** 2021

**Authors:** Maryam Tabarzad, Samaneh Baktash, Vahideh Atabaki, Tahereh Hosseinabadi

**Affiliations:** a *Protein Technology Research Center, Shahid Beheshti University of Medical Sciences, Tehran, Iran. *; b *Department of Pharmacognosy , School of Pharmacy, Shahid Beheshti University of Medical Sciences, Tehran, Iran. *; c *Department of Pharmacognosy and Pharmaceutical Biotechnology, Faculty of Pharmacy , Hormozgan University of Medical Sciences, Bandar Abbas, Iran.*

**Keywords:** Cyanobacteria, Fischerella sp., Mycosporine-like amino acids, UV-absorbing, LC-MS, HPLC

## Abstract

Mycosporin-like amino acids (MAAs) are a group of UV-absorbing compounds, which can be produced by various organisms such as algae and cyanobacteria, particularly if they survive in highly irradiated environments. In this study, the production of MAAs by two species of* Fischerlla* sp. (F5 and F14), isolated from the North of Iran, was investigated. Both species, which had previously been morphologically detected as *Fisherella* sp., were confirmed molecularly by sequencing the PCR amplicon of the *16S rRNA* gene. The species were cultured in sterilized BG.11 medium for 21 days, then biomasses were separated, and their MAAs content was extracted by methanol and partially purified using chloroform liquid-liquid extraction. The extract was analyzed using high-performance liquid chromatography (HPLC) and liquid chromatography-mass spectroscopy (LC-MS). In both species, the compounds with MAAs characteristics were observed. They had maximum absorbance (λ_max_) in the range of 300–400 nm, which was confirmed by the LC-MS analysis. In F5 species, the peaks with m/z 340 and 391 and in another one (F14), a peak with m/z 333.2 were recorded, that the latter might be Shinorine. In general, further analysis should be performed to elucidate the exact structural aspects of these compounds. In conclusion, both *Fischerella* sp. studied here were capable of producing MAAs and can be evaluated for use in sunscreen pharmaceutical and cosmetic products.

## Introduction

Cyanobacteria (blue-green algae) are a group of prokaryotes with photosynthetic properties. Unicellular and filamentous structures are common forms of cyanobacteria (1). They are valuable organisms in aquatic and terrestrial ecosystems. In addition, cyano-bacteria can fix atmospheric nitrogen by converting it into ammonium compounds (2). Cyanobacteria can survive in different habitats and produce various secondary metabolites with valuable and distinctive functions. Among them, photoprotective metabolites such as scytonemin and mycosporine-like amino acids (MAAs) play a remarkable role in their protection against ultraviolet radiation (3). MAAs are colorless and water-soluble small molecules with a cyclohexenone or cyclohexenimine chromophore linked through sulfate esters or glycosides bond to imine functional group of amino acids or amino alcohols (4, 5). Based on the evidence, their biosynthesis takes place through the shikimate and/or pentose phosphate pathways (6). They can absorb light in the range of UV-A and UV-B wavelength (310-362 nm) (7). Due to UV absorption in a broad range of wavelength, high molar extinction coefficient (ε = 28,100–50,000 M^-1^cm^-1^), strong UV-absorption ability, and photostability, MAAs are applied as sunscreen agents in pharmaceutical and cosmetic products and can inhibit skin damage after UV irradiation (5, 8), such as Helioguard 365™ that contains Shinorine and Porphyra-334 (two well-known MAAs) as sun-protective agents (9). MAAs can also have antioxidant properties to scavenge toxic oxygen radicals and protect cells against oxidative stress (10, 11). Several studies have been shown that MAAs could protect cells against DNA damage and could block thymine dimer formation induced by UV irradiation. Moreover, it was found that MAAs promoted the proliferation of human skin fibroblast cells (12). This feature makes MAAs an attractive ingredient in cosmetics.

The presence of MAAs in a number of cyanobacteria species has been studied so far. In addition, the induction of MAAs in different types of cyanobacteria has also been investigated (13, 14). For example, the cyanobacterium *Fischerella muscicola* TISTR8215 was shown to produce UV-absorbing MAAs with maximum absorption at 332 nm and a retention time (Rt) of around 16.1 min, which was induced by UV radiation (13). Accordingly, two species of *Fischerella* sp. was investigated in this study regarding their MAAs production in normal condition (BG11 culture medium, without extra UV irradiation). 

## Experimental

*Identification of cyanobacteria strains*

Cyanobacteria samples (F5 and F14) had collected from the North of Iran. Morphological determination of samples was performed at the Department of Biotechnology, School of Pharmacy, Tehran University of Medical Sciences, Tehran, Iran (15). For molecular confirmation of these two species of cyanobacteria, *16S rRNA* ribosomal gene sequencing was applied.

Cyanobacteria cells (1 mL) were harvested from culture media by centrifugation (SV 1422, Sigma, Germany) at 10000 ×g for 1 min in sterile microtubes. Pellets were suspended in 100 µL of 10% Triton X100, vortexed, and incubated in a dry block incubator (DPI-1T, Kiagen, Iran) at 100 ºC for 60 min. The samples were cooled, vortexed (MS 3 B, IKA, Korea) and centrifuged (10000 ×g, 1 min, and 4 ºC); then, the supernatant of each sample was used as a DNA template. PCR amplification was performed in a total volume 50 µL containing 1 µL of DNA template in Master Mix 2x and 1 µL of general primes (Table 1). The PCR was run in a thermal cycler (T100™, BIORAD, USA) using the following protocol: initial denaturation at 95 °C for 5 min; 35 cycles of 95 °C for 30 s, 57 °C for 30 s, 72 °C for 45 s, and an extension step at 72 °C for 20 min. 

Amplified PCR products were analyzed using 2% agarose gel electrophoresis (Mini horizontal, Clever, UK) in comparison with the 1.5 kbp DNA ladder. Then, the amplified products were purified from agarose gel using the AccuPrep Gel DNA Purification kit (Bioneer, Korea). Finally, the sequences of amplified genes were determined using the standard Sanger method and analyzed using NCBI GenBank and BLAST program.

*Cultivation and determination of growth curve of cyanobacteria*

Both* Fischerella* sp. (F5 and F14) were cultivated in 500 mL Erlenmeyer flask containing 200 mL BG-11 medium and incubated at 25 ºC in germinator under a 16:8 h light/dark program and 70% humidity during 21 days. Two-milliliter culture samples (F5 and F14) were transferred to Falcone tubes containing 20 mL BG-11 and were incubated in the same condition. Then, the cultures were centrifuged at 10000 ×g for 10 min on alternate days, and the obtained biomasses were dried at 60 °C in an oven overnight and then weighed. Growth curves were drawn based on dry biomass weight and growth period using GraphPad Prism.

*MAA extraction from Cyanobacteria*

MAAs were extracted using methanol (MeOH) as described in previous studies (18, 19). Briefly, the cyanobacteria biomasses were separated from 200 mL culture of cyanobacteria by centrifugation at 10000 ×g for 10 min. Pellets were suspended in distilled water and re-centrifuged to remove impurities. These steps were repeated twice and then preserved in a freezer (-80 ºC) overnight and dried using a freeze-dryer (Alpha 1-2 LDplus, Christ, Germany). Then, 10 mL of absolute MeOH were added to 30 mg of dried biomasses. The mixtures were maintained in an ultrasonic bath (1 min) and then refrigerated. After 24 h, the samples were incubated in an ultrasonic bath at 45 ºC for 2 h and then centrifuged (10000 ×g, 15 min, and 4 ºC). Supernatants were collected and dried at room temperature and followed by dissolution in distilled water (1 mL). These samples were centrifuged again, and supernatants were transferred to the new microtubes. After adding 500 µL of chloroform to each sample, they were vortexed and centrifuged (10000 ×g, 10 min, and 4 ºC), and supernatants were collected for HPLC analysis.

*HPLC analysis*

Samples were analyzed by reverse-phase high-performance liquid chromatography (LC-8A, Shimadzu, Japan) equipped with a photodiode array (PDA) detector (SPD-M10A- Shimadzu). 100 µL of each were subjected to HPLC column (C18, 5 µm, 250 × 4.6 mm) through an isocratic mobile system of MeOH (25%) and 0.1% acetic acid with a flow rate of 1 mL/min for 20 min and column eluting compounds were detected in 340 nm.

*LC-MS analysis*

The LC-MS chromatograms of samples were recorded on HPLC (Agilent 1200 series) equipped with Agilent 6410 QqQ mass spectrophotometer with an electrospray ionization (ESI) interface. Samples (200 µL) were injected into the HPLC column (C18, 5 µm) using the same isocratic flow system with a flow rate of 1 mL/min for 10 min. In addition, the absorbance of eluting compounds was recorded at 320 nm.

## Results

*Identification of cyanobacteria genus*

**Figure 1:**
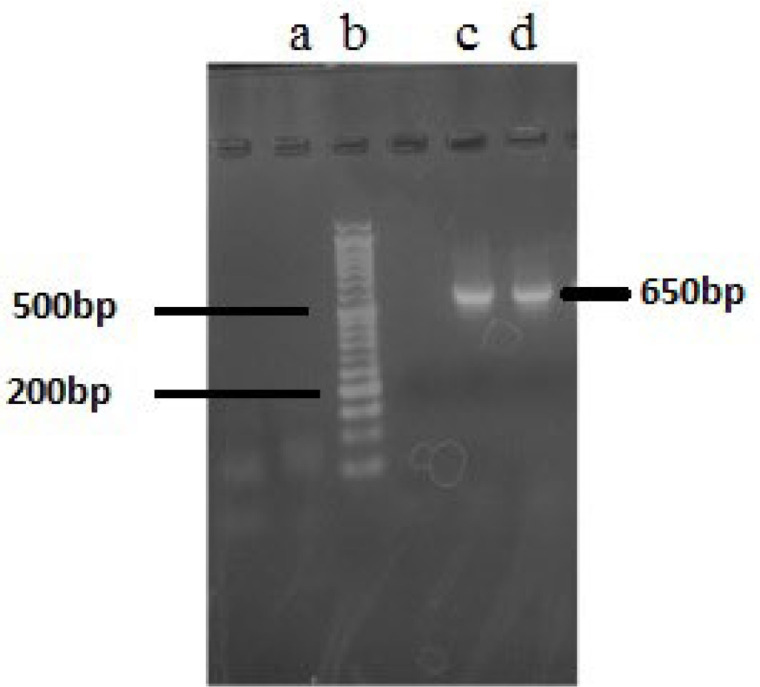
Agarose gel electrophoresis (2%) of *16S rRNA* gene PCR products. (a) blank; (b) 1.5 kb DNA ladder; (c) PCR products of F5; (d) PCR product of F14

Based on the morphological assessment, two cyanobacteria strains (F5 and F14) studied in this research belonged to *the Fischerella* species. However, PCR sequencing of *16S rRNA* genes as a molecular genetic approach was applied for more confirmation. As shown in Figure 1, PCR amplification with two specific primers of CYA106F/ CYAN738R, which results in 650 base pairs amplicon, showed acceptable results. Comparison of gene sequencing results with those available in the NCBI database showed that all of the obtained sequences corresponded to *Fischerella* sp. with high sequence similarity (99.6 %) (Table 2).

*Growth measurements studies*

**Figure 2:**
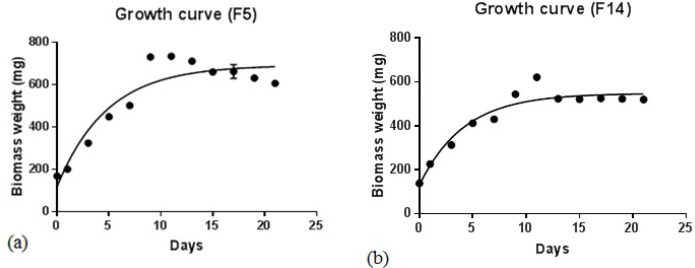
Growth curves of *Fischerella* species, F5 (a) and F14 (b), in BG-11 culture medium at 25 ºC under 16:8 h light /dark condition

*Fischerella *strains (F5 and F14) were cultured in BG11 medium (200 ml), and then growth curves were drawn by the measurement of dried biomasses at 25-day intervals. As shown in Figures 2A and 2B, both species entered the logarithmic phase of growth after 15 days, and then the steady-state phase continued to 25 days. For more production of secondary metabolites, the extraction process was carried out after 21 days.

*HPLC analysis of MAA partial extracts*

Due to the high polarity of MAAs, they were extracted from F5 and F14 species biomass with 100% methanol. The presence of MAAs in these extracts was investigated by HPLC equipped with a PDA detector. In general, mycosporines with a common cyclohexenone ring system and a methoxy moiety at carbon 2 exhibited similar spectral characteristics, which had a maximum absorption (λ_max_) at about 310 nm in H_2_O. Different structures of MAAs which have identical ring-chromophores also had very similar spectral characteristics. Most marine MAAs are imine derivatives of mycosporines containing an amino-cyclohexenimine ring linked to an amino acid, amino alcohol, or amino group and have a maximum absorption between 320 and 360 nm (20). 

**Figure 3:**
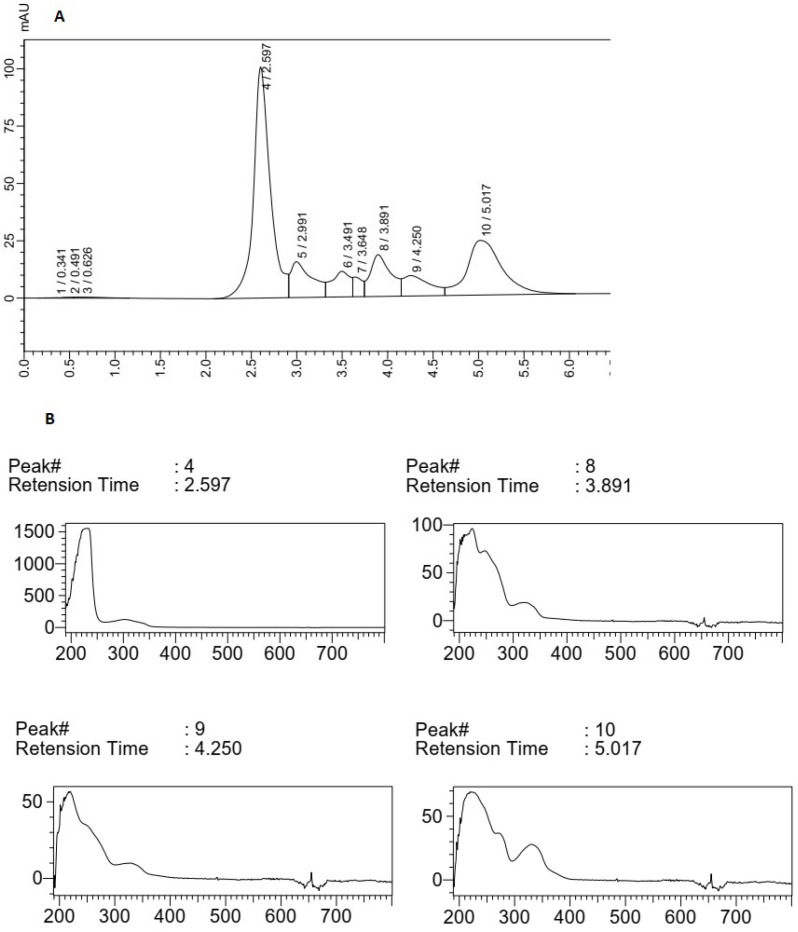
(A) HPLC chromatogram of F5 extract, (B) Peak (4), peaks (8), (9) and (10) correlated with main compounds and the compounds having UV absorption at 300-400 nm

**Figure 4:**
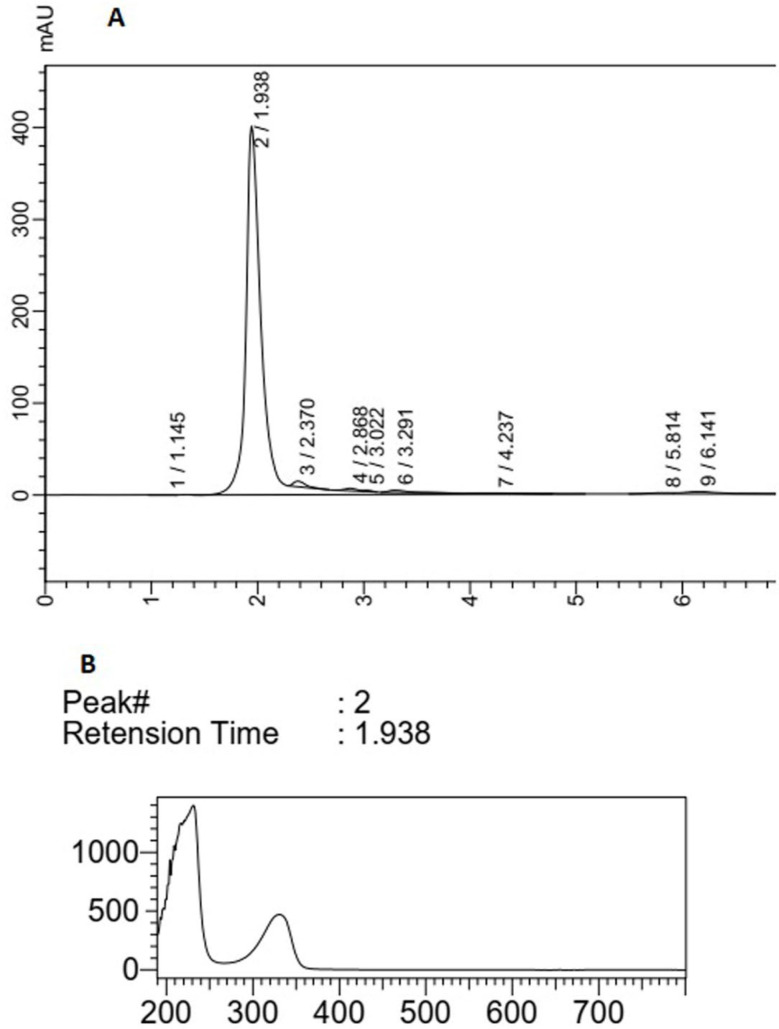
(A) HPLC analysis of F14 extract, (B) peak (2) correlated with compounds having UV absorption at 300-400 nm

The HPLC chromatogram of F5 extract displayed a major peak (Rt of 2.597 min) with maximum absorption of about 270 nm and three peaks (Rt of peaks were 3.89, 4.25, and 5.01 min) with maximum absorption at 310-330 nm, which could show the MAA compounds (Figure 3). The main peak (Rt: 1.93 min) with λ _max_ = 330 nm in the chromatogram of F14 extract could also confirm the production of MAAs in this species (Figure 4). The supernatant of *Fischerella* sp. cultivation mediums was similarly extracted by methanol (100%) and analyzed using HPLC. There was no evidence about any compound with maximum adsorption of 310-360 nm in these samples (data was not presented).

*LC-MS analysis of cyanobacteria extracts*

**Figure 5:**
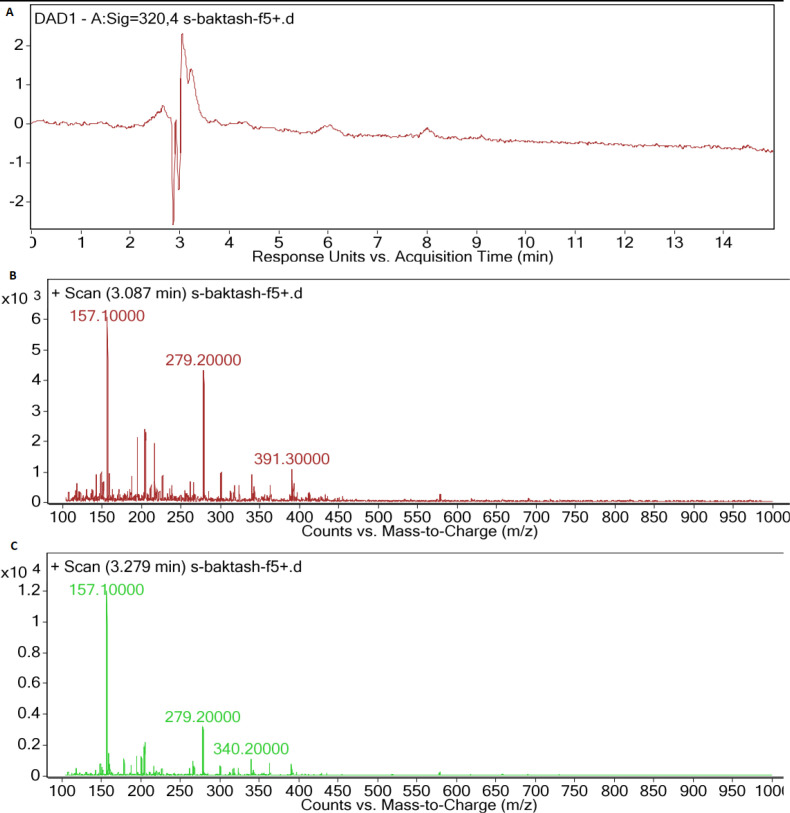
(A) LC-DAD spectra of F5 extract, (B) MS spectrum of main peak (Rt: 3.087 min), (C) MS spectrum of second peak (Rt: 3.279 min), with UV absorption at 300-400 nm

**Figure 6:**
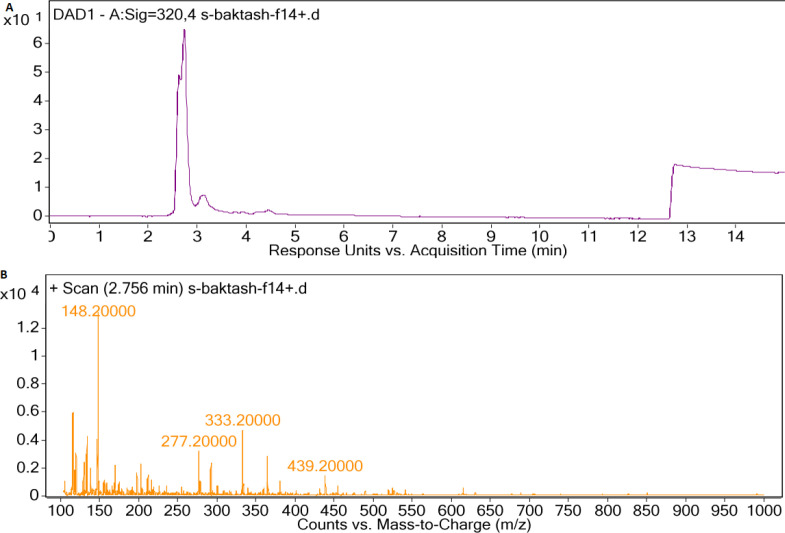
(A) LC-DAD spectra of F14 extract, (B) MS spectrum of main peak with UV absorption at 300-400 nm

HPLC chromatogram of F5 extract displayed peaks at retention times of 3.08 and 3.27 min (Figure 5a), correlated with compounds having [M+H]^+ ^ion peaks at m/z 391.3 (Figure 5b) and 340 (Figure 5c), respectively in the ESI-Mass spectra. Mass spectrum of F14 extract revealed an ion peak at m/z 333, correlated with compound detected at the retention time of 2.75 min in the HPLC chromatogram (Figure 6). Since the most reported MAAs had low molecular weights (< 400 Dalton) (21), these extracted mixtures might contain MAAs or their derivatives.

## Discussion

Several UV-absorbing/screening MAAs have been reported from various organisms, including cyanobacteria (4). However, the production of MAA by different cyanobacteria species still needs to be investigated. In this study, we determined the production possibility of MAAs in two cyanobacteria species isolated from paddy fields of the north of Iran, *Fischerella* sp. (F5 and F14). Studies on MAAs production in *Fischerella* sp. were only limited to *F. muscicola *obtained from Thailand (13).

These isolated cyanobacteria had identified by the morphological study and then confirmed by*16S rRNA* PCR/sequencing analysis here. Methanol (100%) extraction as a common extraction method for partial purification of MAAs (22) was used in this study and then analyzed by HPLC and LC-MS methods. The results of HPLC analysis showed that both *Fischerella* species contained compounds with maximum absorption in the range of 300 to 400 nm. Therefore, it can be inferred that both species have the ability to produce secondary metabolites with MAA characteristics. According to the characteristics, like other MAAs, the extracted MAAs from these *Fischerella* sp. maybe contain a glycine moiety on the C3 and a second group on C1 of the cyclohexenimine ring. The addition of a second amino acid can result in MAAs like Porphyra-334, Shinorine, mycosporine-2-glycine, mycosporine-glycine-glutamic acid. In some MAAs like asterina-330. If and palythinol, the second group was amino alcohol, and in some others like palythene and usujirene, an enaminone system was linked to the C1, which resulted in maximum absorption at 360 357 nm, respectively. There were reported several compounds in which glycine has been replaced by methylamine such as mycosporine-methyl-amine-serine, mycosporine-methylamine-threonine (ʎ_max _= 325-330 nm) or to an amine group like palythine-serine, and palythine-threonine (ʎ_max _= 320 nm) (20).

According to the retention times of the components in HPLC chromatograms, it can be concluded, the extracted compound from F14 is probably more polar than those extracted from F5. However, the major peak of the HPLC chromatogram of F5 extract appeared at the retention time of 2.59 min and had a maximum absorption at about 290 nm, which may be related to the precursors involved in MAA production (23). The λ_max_ of gadusol as the precursor of MAAs was reported at 269 nm (20). Pursuant to a number of studies, the use of stimuli such as UV-A and UV-B radiation, which induced MAA production, may increase the production of MAA-like compounds (24, 25). Therefore, in future studies, it will be considered to increase the MAAs production in these species by UV irradiation. 

In the mass spectra of F5 species, ion peaks at 157, 279, and 391 m/z for one extracted compound, and 157, 279 and 340 m/z for another one were observed. Mass spectra and maximum absorption of the compounds extracted from F5 were in part similar to those of Porphyra (26), Mycosporine-Glycine-Valine (21), or their sugar derivatives. 

In the mass spectra of the compounds extracted from F14 species, an ion peak at 333.2 m/z was observed, which is similar to that of Shinorine (21, 27 and 28). Similarly, Vanessa Geraldes *et al. *showed that out of the 69 cyanobacteria, which were isolated from Brazilian environments, 26 strains could synthesize MAAs. Among them, *Fischerella* sp. (CENA 161) produced Shinorine (29).

The species of F14 produced just one MAA compound; therefore, its purification and isolation may be easier and more cost-effective for using as a single active ingredient in sun-protective products even though extraction of the genes involved in the biosynthesis of Shinorine and its expression in other cyanobacteria can provide a valuable source for its production, same as what was previously performed by Yang *et al.* to develop engineered *Synechocystis* sp. PCC6803 for improved production of MAA (30). 

However, further analysis is required to accurately identify these active metabolites of F5 and F14 *Fischerella *sp. 

**Table 1:** Specific primers for PCR amplification of *16S rRNA* gene

**Amplicon **	**Primer**	**Sequences**	**Tm**	**Site**	**Ref.**
630	CYA106F	CGGACGGGTGAGTAACGCGTG	65.8	106–127	(16)
	CYAN738R	GCTAGGACTACWGGGGTAT	57.5	738–765	
1260	CYAN738F	ATACCCCWGTAGTCCTAGC	57.5	738–765	(17)
	CYAN1281R	GCAATTACTAGCGATTCCTCC	57.8	1281–1302	

**Table 2:** Results of similarity assessment of *16S rRNA* genomic content using BLAST (NCBI).

**Sample **	**Description**	**Max Score**	**Query Cover**	**Per. Ident**	**Accession**
F5	*Fischerella* sp. Sara 1 16S ribosomal RNA gene, partial sequence	928	94%	99.61%	KY618863.1
	*Fischerella* sp. MGCY374 16S ribosomal RNA gene, partial sequence	928	94%	99.61%	KY056817.1
F14	*Fischerella* sp. ATCC 43239 16S ribosomal RNA gene, partial sequence	627	95%	86.52%	KJ768872.1
	*Fischerella* sp. IR-291 16S ribosomal RNA gene, partial sequence	627	95%	86.52%	KY011910.1

## Conclusion

In this investigation, it was proved that both *Fischerella *species collected from the North of Iran are capable of producing MAAs, and therefore, these *Fischerella* species can be further studied and considered as an appropriate source of UV absorbing compounds. Since the culture of cyanobacteria is simple and low cost, they are an attractive source for the natural production of bioactive compounds. In addition, culture optimization and stimuli induction for improving the production of MAA compounds in future studies can result in obtaining a valuable source for the natural production of sun-protective agents. Due to the application of MAAs as valuable compounds in the cosmetics and pharmaceutical industries, further optimization studies in MAAs biosynthesis is highly attractive and important for investment*.*

## Conflict to interest

The authors declare that there are no conflicts of interest. 

## Funding support

This work was financially supported by the Protein Technology Research Center of Shahid Beheshti University of Medical Sciences, Tehran, Iran (Grant# 16512; Ethical code: IR.SBMU.PHARMACY.REC.1398.037). 
